# Repertory on Appendicitis*Prepared for the Kansas State Hom. Society, 1895.

**Published:** 1895-06

**Authors:** W. A. Yingling

**Affiliations:** Nonchalanta, Kansas


### REPERTORY ON APPENDICITIS.*

### INCLUDING TYPHLITIS AND PERITYPHLITIS.

W. A. Yingling, M. D., Nonchalanta, Kansas.

Abdomen. (Compare with Ileo-cæcal Region.)
— alive in right hypochondrium, motion as of something. Inula.
— alive in left hypochondrium, motion as of something. Phos.
— ball, sensation of, rolling in, when turning over on the left side, especially after pus has formed. Lach.
— burning (or heat) in. *Apis*, *Ars.*, Bell., *Bry.*, *Camph.*, Cocc., Colch., Coloc., Crotal-hor., *Doryphora*, Kali-c., Lach., Magn-phos., Merc., Nux-v., Phos., Plat., Plb., Rhus-tox., Sil., Thuj.
— burning in. Compare under Heat, below.
— coldness of. Cocc.
— cold feeling in. Phos.
— constant pain in a limited spot in. Bry.
— crampy, paroxysmal pain beginning close to crest of ilium, right side, stretching to lumbar and hypogastric regions. Diosc.
— cutting pain in a small spot, between umbilicus and right groin. Inula.
— drawing, burning feeling in almost whole of right side of, with a painful, hard swelling in the region from the crest of ilium to middle abdominal line, upward to liver and downward to groin, better gently pressing upward on tumor. Rhus-tox.
— dull throbbing in the. Bry.
— heat in right hypogastric region. Bell., Bry.
— heat and tenderness, can scarcely bear any covering. Crotal-hor.
— heat. See Burning.
— numbness of. Apis.

---

* Prepared for the Kansas State Hom. Society, 1895.

Abdomen, pain in right side of, reaching to right groin and down to scrotum on the same side. Ars.

— pain in right side, severe, knife-like, going through to back. Pyrogen.

— pain in right side, stretching toward liver and into chest. Camph.

— pain in, on awaking, near right anterior superior spinous process of ilium. Card-mar.

— pain severe in lower, as if it would burst, settling finally into ileo-cæcal region. Nitr-ac.

— pain severe, very, on right side of, extending downward toward rectum. Doryphora.

— pains severe, very, in right side of, with distention; pains spread to right inguinal region. Card-mar.

— pain sharp, stitching between umbilicus and right groin. Inula.

— pain sharp in right lower, extending toward right spermatic vessels. Medor.

— pains tensive, contractive, during exacerbation. Cocc.

— pregnancy, quickening of, feeling like the. Thuj.

— pressing in right side of. Prun., Pyrogen.

— pressive pain between navel and groin, worse standing, lying on back or side, inhaling, etc. Arum-mac.

— pressure in right side of, as from a foreign body. Thuj.

— pressure upward from flatulent distention. Phos.

— pulsations in. Acon., Bry., *Card-mar.*, Colch., Lach., Plb.

— rolling-up feeling as of a hard substance in right hypochondrium. Op.

— rumbling in. Bapt., Carb-sulf., Doryphora, Inula, Nitr-ac., Op., Phos., Plb., Rhus-tox.

— rumbling in, as if a boiler were working in bowels. Nitr-ac.

— rumbling in, caused by pressure. Diosc.

— sensitiveness to contact, extreme. *Lach.*, Plb., Pyrogen.

— sensitive to pressure or touch (tenderness). Apis, Arn., Arum-mac., Bapt., Bry., Camph., Carb-sulf., Colch., Crotal-hor., Diosc., Doryphora, Natr-sulf., Nitr-ac., Plb., Pyrog.

Abdomen, soreness of right side of. Pyrog., Zing.
— soreness of walls of. Bell., Thuj.
— sore pain on a small place on right side. Zing.
— stitches from, into chest. Bry.
— squeezing pains in different parts of, paroxysmal, coming on during quiet or motion. Natr-sulf.
— surface of, hotter than the rest of the body. Colch.
— swollen, distended. Acon., Apis, Ars., Bapt., Bry., Carb-sulf., Card-mar., Colch., Doryphora, Nitr-ac., Phos., Rhamnus-cath.
— tympanitic. See Tympanitis.
Abscess, deep-seated. Apis, Ars., Graph., Hepar, Iod., Kali-c., Lach., Lyc., Merc., Sil., Sulph.
Alive, painful motion in right hypochondrium, as from something. Inula.
— painful motion in left hypochondrium, as from something, when standing or sitting. Phos.
Anxiety, with. *Acon.*, *Ars.*, Bell., Bry., Camph., Coloc., Hepar, Kali-c., Lach., *Merc.*, Natr-sulf., *Nux-v.*, Op., Phos., Plat., *Plb.*, Rhus-tox., Sil., Thuj.
Appendicitis, especially. Apis, Arn., Ars., Arum-mac., Bapt., Bell., Bry., Camph., Carb-sulf., Card-mar., Cocc., Colch., Coloc., Comoclad., Crotal-hor., Diosc., Doryphora, Ginseng, Hepar, Hura, Inula, Lach., Medor., Merc., Merc-cor., Natr-sulf., Nitr-ac., Op., Phos., Plb., Rhamnus-cath., Rhus-tox., Thuj.
Appendix, extreme pain in region of. Crotal-hor.
Appetite, loss of. Crotal-hor.
— Consult general repertory.
Back, forced to lie on, motionless. Bell.
— lying on, with right leg flexed or elevated. Amel., Rhus-tox.
— lies on, with right knee flexed. Hepar, Lach., Merc.
— lying on, aggravates. Acon., *Arum-mac.*, Ars., Coloc., *Nux-v.*, Phos., Rhus-tox., Sil.
Belching. See Eructations.
Bladder, pressure on. Op.

Breathing, aggr. from. Acon., Arum-mac., Bell., *Bry.*, Camph., Carb-sulf., *Cocc.*, Colch., Doryphora, Hepar, Kali-c., *Merc.*, Pyrogen, Rhus-tox., Thuj.

Burning in abdomen. See Abdomen.

— pains. Ars., Bry., Doryphora, Phos.

Chilliness, with. Hura.

Coldness, attacks of. Hepar.

Colic, with anxiety. Arum-mac.

— from incarceration of gases. Carb-sulf.

— or griping pains. Rhamnus-cath.

Colicky pains in right side. Card-mar.

Collapse, threatened with. Ars., Camph., Crotal-hor., Lach., Verat.

Colon, transverse, aching, sore, bruised feeling felt along the, Merc-cor.

Constipation, with. Bry., Card-mar., Crotal-hor., Lach., Merc., Op., Plat., Plb.

— See also under Stool, and consult general repertory.

Contractive pains. Cocc.

Coughing, aggr. from. Arn., Ars., Cocc., Plb., Pyrogen.

Covering, throws off, though the body is cold to the touch. Camph.

Crawling sensation extending to the toes, distressing. Ginseng.

Cutting pains. Ars., *Bell.*, Bry., Carb-sulf., Card-mar., Colch., *Coloc.*, Comoclad., Crotal-hor., *Diosc.*, Inula, Kali-c., *Merc.*, Nux-v., Op., Plat., Rhamnus-cath., Rhus-tox., Sil., Thuj.

Delirium when going to sleep, with. Ginseng.

Diarrhœa. See under Stool, and consult general repertory.

— alternating with constipation, copious, gushing, exhausting stool. Phos.

— bilious. Apis.

— involuntary, thin, offensive, or retention of stool. Op.

Drawing pains. Bry., Camph., Card-mar., Hepar, Inula, Kali-c., Lach., Medor., Merc., Nux-v., Plat., Plb., Rhus-tox., Thuj.

Drinking, pain aggr. from. Doryphora.

Driving, aggr. while. Card-mar.
Dull pain. Bry.
Eating, pain worse from. Doryphora.
Enteritis from pressure of foreign bodies. Bry.
Erections, frequent. Plat.
— with ineffectual desire for stool. Thuj.
Eructations, acrid, bitter, loud. Carb-sulf.
— many, but do not relieve. Op.
— putrid. Arn.
— sour. Plb.
Extremities, cold. Op.
— constant motion of. Cocc.
— See Legs.
Exudation. Apis.
— purulent. Merc.
Eyes, half open. Op.
— obscuration of sight. Op.
Face, anxious. Rhus-tox., Plb.
— cold and pale. Hepar.
— cold sweat on. Ars.
— flushed or pale. Merc.
— hippocratic. Camph., Op.
— hot. Hura.
— pale. Merc., Op., Rhus-tox.
— troubled look in. Plb.
Fainting spells. Camph., Carb-sulf.
— with nausea. Carb-sulf.
Feet, caused by getting feet wet. Rhus-tox.
— cold. Ars., Hura.
— damp. Hura.
— odœma of the. Apis.
Flatus, better passing. Arn.
— fetid. Arn., Ars., Camph., Carb-sulf., Cocc., Nux-v., Phos., Sil.
— free discharge of. Arn., Carb-sulf., Phos.
— incarcerated. Carb-sulf., Natr-sulf., Phos.
— odorless. Phos.

Flatus, sour. Carb-sulf., Merc.

Fluid in ileo-cæcal region, sense of, on pressure. Apis.

Food, sight or smell of, causes nausea and aversion. Colch.

Fermentation. See Rumbling, under Abdomen.

Fever, with. Bell., Ginseng, Lach.

— after sleep. Lach.

— at 3 P. M. Lach.

— Consult general repertory.

Gnawing pains between spine of ilium and rectus muscle. Medor.

Griping pains. Inula, Op., Phos., Rhamnus-cath.

Groin. Consult Ileo-cæcal Region.

— drawing pains in right and over external pubic region. Inula.

— outward pressure in, with rolling, rumbling, and distention. Natr-sulf.

— pains beginning in right and spreading over abdomen. Natr-sulf.

— pains extend to. Ginseng.

— pressure in, as from a foreign body. Thuj.

— stitching pain in right. Bapt., Inula.

— stitching pain in right extending to umbilicus with each step. Inula.

Hands, burning of palms. Rhus-tox.

— cold. Ars.

Head, aching of the. Plb.

— confusion of the. Op.

Heat, after sleep. Lach.

— when going to sleep. Ginseng.

Hernia-like pains. Cocc.

Hiccough. Op., Phos., Nux-v.

Hypochondrium. See under Abdomen.

Ileo-cæcal region, (right iliac fossa). Compare with Abdomen.

— Bapt., Bry., Carb-sulf., Diosc., Ginseng, Magn-phos., Merc-c., Phos., Plb., Thuj.

— bruised, sore feeling in, sensitive to pressure. Merc-c.

— circumscribed tumor in, the size of a large turnip, yielding and yet hard to the touch. Coloc.

Ileo-cæcal, cutting, griping pains in. Rhamnus-cath.
— cutting, tensive pains in, worse from deep inspiration. Thuj.
— deep, circumscribed swelling in. Hepar.
— feeling of hardness over cæcum, with severe pain. Crotal-hor.
— gurgling, rumbling in. Apis, Carb-sulf., Ginseng, Natr-sul., Rhamnus-cath.
— indurated. Apis, Magn-phos., Merc., Rhus-tox., Plb.
— pain in, reaching to groin and scrotum on same side. Ars.
— painful, hard, hot, red swelling in. Merc.
— sensation of fluid, present on pressure. Apis.
— sensitive to pressure (tenderness). Arn., Bapt., Cocc., Colch., Crotal-hor., Ginseng, Lach., Merc-c.
— sensitive to touch (painful). Apis, Arn., Bell., Bry., Card-mar., Colch., Crotal-hor., Merc., Merc-c., Nitr-ac., Plb.
— sharp, severe pains in. Bell., Gingseng, Hura, Magn-phos.
— steady, unremitting pain in. Cocc.
— sudden pain arising in a small area in, suddenly ceasing in vomiting or headache. Diosc.
— swelling like a tumor in, tensive, drawing pain in. Medor.
— swelling like a tumor in, hard, worse least motion, touch, sneezing, coughing. Plb.
— swollen. Apis, Arn., Card-mar., Colch., Ginseng, Hepar, Lach., Magn-phos., Medor., Merc., Natr-sulf., Phos., Plb., Rhus-tox.
— tenderness on pressure at a small spot the size of an orange, great, some feeling of hardness. Crotal-hor.
— twitching, cramping pains in, which spread over the whole right side of abdomen. Carb-sulf.
Inguinal region, outward, pressure at right. Bell.
— tenderness. See Abdomen and Ileo-cæcal Region.
Inspiration. See Breathing.
Intermittent pains. Bapt., Comoclad., Crotal-hor., Diosc., Inula, Natr-sulf.
Intestines, increased peristaltic action of, with rumbling in cæcum. Carb-sulf.
— paralysis of. Phos.

Jar, least, aggr. Acon., *Arn.*, *Bell.*, Bry., Hepar.

Knees drawn up, must have the. Crotal-hor., Hepar, Lach., Merc., Op., Rhus-tox.

Lancinating pains. See Cutting Pains.

Legs, aching of the. Plb.

— cold, clammy sweat on. Plb.

— draw up the, must, abdomen hard and tympanitic. Op.

— extending or moving right, aggr. Rhus-tox.

— extending the right, greatly aggravates the pain, must lie with it drawn up and propped with a pillow. Crotal-hor.

— lame feeling in the. Plb.

— lancinating pains radiating down the right, with numbness in it. Plat.

— pains from rotating the right. Lach.

Lie on back. See Position and Back.

Loin, pain in right, with tense feeling. Natr-sulf.

— tense feeling, painful stiffness, from right into sacrum, groin, and anterior part of thigh. Lach.

Mercury, ill-effects from. Hepar.

Mesenteric tuberculous deposits. Hepar.

Motion, aggr. from. Apis, Arn., *Bell.*, *Bry.*, Camph., Cocc., Colch., Hepar, Merc., *Natr-sulf.*, Nux-v., Phos., *Plb.*, Pyrogen, Sil.

— during, sharp stitches in ileo-cæcal region. Hura.

— painful feeling of, in right hypochondrium, as from something alive. Inula.

Nausea, with. *Ars.*, Bapt., *Bell.*, Bry., Carb-sulf., Card-mar., *Cocc.*, Colch., Diosc., Hepar, Hura, Merc., Natr-sulf., Nux-v., Phos., Plat., *Plb.*, Rhus-tox., Sil.

— from sight or smell of food. Ars., *Colch.*

— from stooping. Carb-sulf.

Nose, worse blowing the. Arn.

Pains come and go rapidly. Bell., Magn-phos.

— crescendo and diminuendo in character. Bell.

— crescendo in character. Diosc.

— doubling up and extorts cries, causes. Coloc.

— extending downward toward rectum. Doryphora.

Pains extending and following downward direction of rectus muscle. Comoclad.
— internal, more. Camph.
— itching character, of an. Carb-sulf.
— of inflammation. Natr-sulf.
— pressive, about quadratus lumbar muscle, worse when rising from the lying posture. Rhus-tox.
— rotating the right leg, when. Lach.
Paroxysmal pain. See Intermitting Pain.
Peritonitis. Phos.
Perityphlitis. See Typhlitis.
Perspiration. See Sweat.
Pinching pains. Cocc., Phos.
Position, impossible to lie on left side. Rhus-tox.
— lies on back with the right knee flexed. Hepar, Lach., Merc., Rhus-tox.
— lies motionless on back, must. Bell.
Pressing aggravates or causes pains to return. Carb-sulf.
— pains. Apis, Arn., Ars., Bell., Camph., Card-mar., Cocc., Colch., Merc., Nux-v., Phos., Pyrogen, Rhus-tox.
— swelling from below upward, amel. the pain. Rhus-tox.
Pressure, causes rumbling in right side of abdomen. Diosc.
— outward at right inguinal region. Bell., Cocc., Natr-sulf.
— sensitive to. Apis, Arn., Bapt., Bell., Bry., Camph., Carb-sulf., Cocc., Colch., Crotal-hor., Diosc., Doryphora, Ginseng, Lach., Merc., Merc-c., Natr-sulf., Nitr-ac., Plb.
— sensitive to. See also Abdomen and Ileo-cæcal Region.
Prostration, with great. Camph., Crotal-hor., Diosc., Nitr-ac.
Pulse, hard and tense. Op.
— rapid. Camph., Crotal-hor., Rhus-tox.
— slow. Op.
— weak. Camph., Crotal-hor., Rhus-tox.
— Consult general repertory.
Quiet, better from. See Motion, worse from.
— pains coming when, and also when moving. Natr-sulf.
Rectum, pressure on the. Op.
Remits, when the pain remits it returns intensified. Cocc.

Restlessness, with. Acon., Ars., Bell., Cocc., Coloc., Natr-sulf., Pyrogen.
— Consult general repertory.
Retching, with. Ars., Bapt., Hepar, Plb.
Rheumatic subjects, in. Bry.
Rumbling. See under Abdomen.
Septic states, in. Crotal-hor., Pyrogen, Rhus-tox.
Sharp pains. Arn., Bapt., Bell., Coloc., Ginseng, Inula, Magn-phos., Medor., Phos.
Shooting pains. Phos. (Consult Sharp Pains.)
Side, lying on, aggr. Arum-mac.
— numerous stitches in right, worse while lying on painful side. Thuj.
— pressing pain on right, between false ribs and hips, worse when stretching out the body in the morning, with colicky pains. Card-mar.
Sight. See Eyes.
Sitting, worse when. Phos., Rhus-tox.
Skin, hot and dry. Crotal-hor.
— pale, cold, and clammy. Diosc.
— perspiring while it burns to the touch. Bell., Op.
Sleep, restless. Inula.
— starting and crying out during. Inula.
Sleeplessness. Bell.
Sleepy, but cannot sleep. Bell.
— and stupid. Op.
Sneezing, aggr. from. Apis, Plb.
Spasmodic pains. Cocc.
Squeezing pains. Natr-sulf., Op.
— pains, as if something were forced through a narrow space. Op.
Stages of the disease, first. Acon., *Bell.*, Merc.
— suppurative. Hepar, Merc.
— suppurative. See Abscess.
— typhoid. Apis, Bapt., Bell., Bry., Hepar, Lach., Merc., Merc-c., Plb., Pyrogen, Verat.
Standing, worse. Arum-mac., Bry., Phos.

Stepping, worse from. See Walking.
Stercoraceous vomiting. See Vomiting.
Stinging pains. Apis, Bry., Camph., Ginseng.
Stitching pains. Ars., Bapt., Bry., Colch., Hura, Inula, Merc., Thuj.
— pressive, cutting, from right to left, worse walking. Merc.
Stomach, coldness of. Cocc.
— painful distention of. Phos.
Stool, almost incessant desire for. Merc-c.
— constipation, or slimy, difficult. Merc.
— copious, gushing. Phos.
— curdy masses or pus. Lach.
— fetid. Crotal-hor., Op.
— frequent calls to. Arn., Hepar, Nux-v.
— ineffectual desire for. Inula, *Nux-v.*, Thuj.
— involuntary. Op.
— mucous. Merc., Merc-c., Nitr-ac.
— painful urging to. Lach.
— retention of, or involuntary, offensive, thin diarrhœa. Op.
— scanty of blood and mucus. Merc-c.
— watery. Nitr-ac.
— whitish, containing pus. Rhus-tox.
— See also Constipation.
Suppurative. See Abscess.
Sweat, cold. Ars., Hura, Plb.
— cold on face. Ars.
— cold on forehead. Plb., Verat.
— copious upon genitals, of a honey-like odor. Thuj.
— on uncovered, while covered parts are hot. Thuj.
— profuse at night. Rhus-tox.
— with. Camph., Op., Plb.
— with the pains. Merc.
Tearing pains. Ars., Calendula, Colch.
Temperature, subnormal. Crotal-hor., Heloderma.
Tenderness. See under Abdomen and Ileo-cæcal Region.
Tensive pains. See Drawing Pains.
Testicle, tenderness of the right. Medor.

Thirst, with great. *Ars.*, Bell., *Bry.*, Cocc., Crotal-hor., Merc., Nux-v., Op., Plb.
— great for large quantities. Bry.
— great for small quantities. Ars.
Thirstlessness. Apis.
Throbbing. Bry.
Tongue, brown streak in centre. Apis, Plb.
— black. Phos.
— cracked and glossy. Phos.
— dry. Apis, Ginseng, Merc., Phos., Plb., Rhus-tox.
— foul. Crotal-hor.
— large, shining papillæ, with. Ginseng.
— red. Merc., Phos., Rhus-tox.
— red at tip. Crotal-hor., Rhus-tox.
— red edges. Plb., Pyrogen.
— sides moist. Apis.
Tossing about, agonizing. Coloc.
Touch. See Pressure.
Traumatism, from. Apis, Arn., Bry.
Twitching pains. Carb-sulf.
Tympanitis, with. Apis, Arn., Ars., Diosc., Op., Phos., Rhamnus-cath.
— Also consult general repertory.
Typhlitis (especially). Acon., Bell., Bry., Card-mar., Colch., Crotal-hor., Crot-tig., Diosc., Ginseng, Hepar, Lach., Lyc., Merc., Nux-v., Op., Plat., Plb., Pod., Rhamnus-frang., Rhus-tox., Thuj.
Umbilicus, drawn in. Plb.
— retracted, with hiccough. Op.
— See under Abdomen.
Unremitting, steady pains. Cocc.
Urine, dark. Apis, Lach.
— offensive mucous discharge, during the flow. Lach.
— scanty. Apis, Lach.
— secretion diminished. Apis.
— urging to, too frequent. Hepar.
— with red sediment. Lach.

Vision. See Eyes.
Vomiting, with. Acon., Apis, Ars., Bapt., Bell., Bry., Carb-sulf., Colch., Coloc., Diosc., Hepar, Natr-sulf., Op., Phos., Plb., Plat., Sil.
— after eating or drinking. Ars.
— as soon as water becomes warm in the stomach. Phos.
— bilious, bitter. Apis, Ars., Bry., Carb-sulf., Coloc., Crotal-hor., Hepar, Merc., Phos., Plb.
— disposed to, when sitting up. Colch.
— fatty. Thuj.
— grass-green substance. Rhus-tox.
— offensive. Ars., Cocc., Nux-v., Op., Phos.
— sensation as if vomiting would do good. Bapt., Nux-v.
— sensation as if he would vomit, without nausea. Bapt.
— stercoraceous. Acon., Ars., Bell., Bry., Lyc., Merc., *Nux-v.*, *Op.*, *Plb.*
— yellow, green mucus. Phos.
Walking, worse from, Arn., Hura, Inula, Merc., Rhus-tox.
Wandering pains (Abd.). Card-mar., Comoclad.
Weakness, with. See Prostration.
Zymotic states, in. Crotal-hor., Pyrogen.

